# Molecular Identification of *Trypanosoma evansi* Isolated from Arabian Camels (*Camelus dromedarius*) in Riyadh and Al-Qassim, Saudi Arabia

**DOI:** 10.3390/ani11041149

**Published:** 2021-04-17

**Authors:** Dina M. Metwally, Isra M. Al-Turaiki, Najwa Altwaijry, Samia Q. Alghamdi, Abdullah D. Alanazi

**Affiliations:** 1 Department of Zoology, College of Science, King Saud University, Riyadh 11451, Saudi Arabia; 2 Department of Parasitology, Faculty of Veterinary Medicine, Zagazig University, Zagazig 44519, Egypt; 3 Department of Information Technology, College of Computer and Information Sciences, King Saud University, Riyadh 11451, Saudi Arabia; 4 Department of Computer Science, College of Computer and Information Sciences, King Saud University, Riyadh 11451, Saudi Arabia; ntwaijry@ksu.edu.sa; 5 Department of Biology, Faculty of Science, Al-Baha University, Al-Baha 1988, Saudi Arabia; sqassim@bu.edu.sa; 6 Department of Biological Science, Faculty of Science and Humanities, Shaqra University, Ad-Dawadimi 11911, Saudi Arabia; aalanazi@su.edu.sa; 7 Department of Medical Laboratory, Alghad International Colleges for Applied Medical Science, Tabuk 47913, Saudi Arabia

**Keywords:** *Trypanosoma* spp., camel, ITS-1, Saudi Arabia, Surra, polymerase chain reaction (PCR), thin blood smear

## Abstract

**Simple Summary:**

We investigated the occurrence of the parasite *Trypanosoma evansi* in camels in Saudi Arabia. Despite being undetectable in thin blood smears obtained from the camels, polymerase chain reaction findings show that nearly half the camels were infected with parasites. Infection causes a significant financial burden to camel breeders and owners. Detection of the parasite reduces financial losses and improves camel mortality. We conclude that polymerase chain reaction is more effective than microscopy at identifying *T. evansi* infection in camels.

**Abstract:**

We analyzed the blood from 400 one-humped camels, *Camelus dromedarius* (*C. dromedarius*)*,* in Riyadh and Al-Qassim, Saudi Arabia to determine if they were infected with the parasite *Trypanosoma* spp. Polymerase chain reaction (PCR) targeting the internal transcribed spacer 1 (ITS1) gene was used to detect the prevalence of *Trypanosoma* spp. in the camels. *Trypanosoma evansi* (*T. evansi*) was detected in 79 of 200 camels in Riyadh, an infection rate of 39.5%, and in 92 of 200 camels in Al-Qassim, an infection rate of 46%. Sequence and phylogenetic analyses revealed that the isolated *T. evansi* was closely related to the *T. evansi* that was detected in *C. dromedarius* in Egypt and the *T. evansi* strain *B15.1 18S* ribosomal RNA gene identified from buffalo in Thailand. A BLAST search revealed that the sequences are also similar to those of *T. evansi* from beef cattle in Thailand and to *T. brucei B8/18* 18S ribosomal RNA from pigs in Nigeria.

## 1. Introduction

*Trypanosoma evansi* is a protozoan parasite of both the intra- and extra-vascular fluids of mammals that causes the disease Surra throughout the tropical and subtropical regions of the world [1]. *T. evansi* has a varied host range that includes many animals, and it is especially prevalent in horses and camels. It is transmitted mechanically by the hematophagous flies *Tabanus, Chrysops, Atylotus, Lyperosia, Haematopota*, and *Stomoxys*. The disease, which can be fatal, causes decreased milk production, anemia, weight loss, and abortion [2,3]. It is broadly distributed in Africa, Asia, Central and South America, the Canary Islands, and recently, in France and Spain [2]. Surra is a serious disease that is difficult to treat and for which there is no vaccine. It is the most dangerous protozoan disease that affects camels, and it can lead to widespread economic losses. In Saudi Arabia, which has a camel population estimated at nearly 1.3 million, these losses can be staggering. Thousands of live camels are imported yearly from Arabian Gulf countries, Somalia, Sudan, and Djibouti [4]. The camels, which are mainly used as pack animals and as sources of meat, milk, skin, and leather, play an important role in transportation in the desert and rural lifestyles. Previous epidemiological, serological, biochemical, and hematological studies have been conducted in camels from Saudi Arabia [5,6,7,8,9,10]. However, there is little information on trypanosomiasis infections in Saudi animals at the molecular level [11]. Based on trypanosomal DNA detection in blood samples, polymerase chain reaction (PCR) testing is a good method for diagnosis of trypanosomiasis. PCR is adaptive and sensitive enough to identify the infection in all phases [12].

Several sequences, such as ribosomal DNA, internal spacer transcribed region (ITS), kinetoplast DNA, and variable surface glycoprotein (VSG), are reliable *T. evansi* detection targets [10,13,14,15,16,17,18,19,20,21]. ITS-1 and ITS-2 are useful for species delineation and inference of *Trypanosoma* spp. phylogenetical relations [22,23], particularly when morphological characters are limited. Phylogenetic analysis of ITS sequences was used in [24] for the study of *T. rangeli* polymorphism. In *T. brucei* [25], *T. congolense* [26], and *T. evansi*, genetic variation in intra-species Trypanosoma has already been identified using PCR-based approaches. The goal of this study is to use internal transcribed spacer 1 primers (ITS-1) to investigate and distinguish the genetic relationship between isolates of *T. evansi* from camels in Riyadh and Al-Qassim, Saudi Arabia.

## 2. Materials and Methods

### 2.1. Ethical Statement

This study (IRB number: SH 11-2020) was approved by The Official Ethics Committee of the Faculty of Sciences and Humanities, Shaqra University, Saudi Arabia.

### 2.2. Study Area

This study was performed in two separate regions of Saudi Arabia: the central region (around Riyadh) and the Emirate of Al-Qassim Province. Riyadh city (latitude: 24°380 North, longitude: 46°430 East) is located in the central region of the Kingdom of Saudi Arabia. It has an exceptionally dry climate; summer temperatures rise during the day but drop at night, changing drastically from to 43 °C to 35 °C. During the winter months, the temperature drops severely and may reach as low as 0 °C. Riyadh is 600 m above sea level with a few valleys and resource-rich sand hills. The Emirate of Al-Qassim Province is located in the heart of the country near the geographic center of the Arabian Peninsula. It extends between 26.2078° North and 43.4837° East, and it has a typical desert climate. The Emirate of Al-Qassim Province is known for its cool, rainy winters and hot summers, which are less humid than those of Riyadh.

### 2.3. Collection of Camel Blood Samples

During the period between June 2020 and January 2021, 400 Arabian Camels (*C. dromedarius*) were randomly selected as follows: 200 camels from Riyadh (67 males, 133 females) and 200 camels from Al-Qassim (71 males, 129 females); camels 4 years and older were considered to be adults. All camels were examined for trypanosomiasis. Blood samples were collected in Vacutainer tubes containing Ethylene Diamine Tetra Acetic acid (EDTA) (BD Vacutainer^®^ Tube, Gribbles Pathology, VIC, Australia) and then divided into two parts. One part was used for microscopy examination, and the other was preserved at −20 °C for the extraction of the DNA of trypanosomes for PCR. The samples were transported to the Parasitological Laboratory of the Biological Sciences Department, Faculty of Technology and Humanities, Shaqra University. For microscopy examination of samples, thin blood smears were air-dried and fixed in methanol. Samples were then stained in a phosphate-buffered saline with a 10% solution of Giemsa (pH 7.2) for four hours in order to perform parasitological analysis. Samples were then analyzed by light microscopy at a magnification of ×40 with an oil immersion objective [27].

### 2.4. DNA Extraction and Polymerase Chain Reaction (PCR)

Total genomic DNA (gDNA) was isolated using the DNeasy Blood & Tissue Kit (250) (Cat No./ID: 69506, Qiagen, Hilden, Germany) and 50 µL or 100 µL of buffer, as dictated by the manufacturer’s protocol. The 250–270 bp region of ITS-1 was amplified using the primer pairs described in Table 1 [16] and a thermal cycler (Variety^®^ 96-well thermal cycler, model 9902, Biosystems, Waltham, MA, USA). The Primer genes are specific to Trypanosoma-related parasites and subspecies, and amplifying a 250 bp, 400 bp, 480 bp and 700 bp for the regions of ITS-1 of *T. vivax*, *T. simiae*, *T. brucei,* and *T. congolense* savannah, respectively [16,28]. This process was performed in 50 µL of a mixture containing 25 µL of DreamTac DNA polymerase Master Mix 2× (Thermo Scientific ™, UK), 1 µL of each primer, and 2 µL of DNA template. The reaction was brought to a total volume of 50 µL with PCR-grade water (Invitrogen, UK). All tests included positive and negative controls. The PCR program consisted of a denaturing step of 95 °C for 2 min, followed by 40 cycles of denaturing at 95 °C for 30 s, primer annealing at 58 °C for 30 s, and 72 °C for 1 min for extension and final extension step was performed at 72 °C for 5 min. Samples were kept at 4 °C. The PCR product was analyzed by 1.5% agarose gel electrophoresis that included 10 μL/mL Syber safe (Thermo Scientific™, UK), which had a trace acetate EDTA buffer at 100 V for 45 min and photographed with a UV imaging system (Imagconat Laz4000, GE Healthcare Life Sciences, UK). The size of each product was estimated by comparing it with a 100 bp gene ruler DNA ladder marker (Thermo Scientific UK, UK) [11].

### 2.5. Nucleotide Sequences of the ITS-1 Gene and Phylogenetic Tree Analysis

To verify the presence of *T. evansi*, positive samples were posted to Macrogen Europe (Netherlands) for sequencing of the ITS-1 region using forward ITS-1 CF and reverse ITS-1 BR primers. The results were compared with existing sequences from the NCBI GenBank. The sequences were processed using Geneious Prime Build 07-04-2020 08:42 [28]. As a pre-processing step, all sequences underwent truncation using an error probability method with a limit of 0.05 on both sides. Related sequences were retrieved from GenBank after performing a BLAST [29] search. Multiple sequence alignments were generated using CLUSTAL Omega [30] implemented in the Geneious software. The phylogenetic tree was created using the neighbor-joining method [31] with a Tamura_Nei model and 10,000 replicates.

### 2.6. ID of Query Sequence

All sequences were deposited in Genbank and assigned accession numbers. Riyadh samples: MW598372, MW579327, and MW579326. Qassim samples: MW598374, MW598373, MW580859, and MW580858.

### 2.7. Statistical Analysis

All data were recorded and filtered in an MS Excel spreadsheet before being analyzed using SPSS version 20. Feedback statistics were used to calculate the spread of the infection. Chi-square was used to compare prevalence of *T. evansi* between regions. Results were considered significant at *p* ≤ 0.05.

## 3. Results

### 3.1. Blood Smear Examination and PCR Amplification of T. evansi

**Figure 1:**
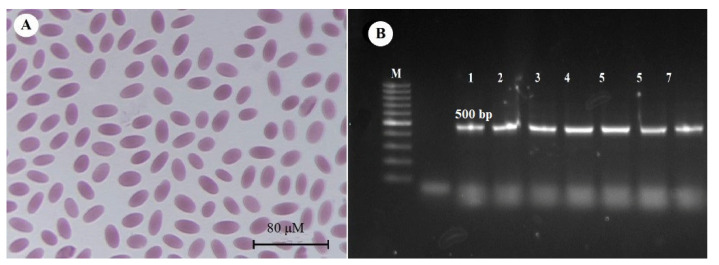
(**A**) Blood smear from camel was free of visible infection with *Trypanosoma* spp. (**B**) Agarose gel (1.5%) electrophoretogram with 100-bp DNA ladder. PCR analysis of the ITS-1 gene revealed a 500 bp band derived from *T. evansi* isolates from different camels (lanes 1–7).

*Trypanosoma evansi* infections were not microscopically observed in the thin blood smear samples from Riyadh or Al-Qassim province (Figure 1A). However, the PCR-positive rates for *T. evansi* in blood samples from Riyadh and Al-Qassim province were 39.5% and 46%, respectively (Table 2). PCR results with ITS-1 primers revealed a molecular weight of 500 bp (Figure 1B). The prevalence of *T. evansi* in young male camels from Riyadh and Al-Qassim was 54.5% and 60%, respectively; the prevalence in adult males was 37.8% and 37%, respectively. The infection rate in young female camels was 40.4% and 60%, respectively, and in adult female camels from Riyadh and Al-Qassim, it was 35.8% and 50%, respectively (Table 3). It was found that the sex and age of the animals had no significant effect on the prevalence of infection in either location.

### 3.2. Nucleotide Sequence Analysis of the ITS-1 Gene

The pairwise identity of Riyadh sequences was 97.9%, while it was 97.6% for the Qassim sequences. Overall, the seven sequences shared 95% pairwise identity and had a guanine-cytosine (GC) content of 42.4%.

A BLAST search showed that the sequences are similar to *T. evansi* SANTE-FANO-HS, isolated from ticks infesting *Canis lupus familiaris* in Egypt (MG564285), *T. evansi* genes for *18 S ribosomal RNA* identified in Egypt from dromedary camels (*C. dromedarius*) 

(AB551919-AB551922), *T. evansi* strain *B15.1 18 S ribosomal RNA* gene identified in Thailand from buffalo (AY912271-AY912276), *T. evansi* from beef cattle (LAM19) in Thailand (EF545999-EF546045), and *T. brucei B8/18 18S ribosomal RNA* genes from pigs in Thailand and Nigeria (AF306772-AF306777).

**Figure 2:**
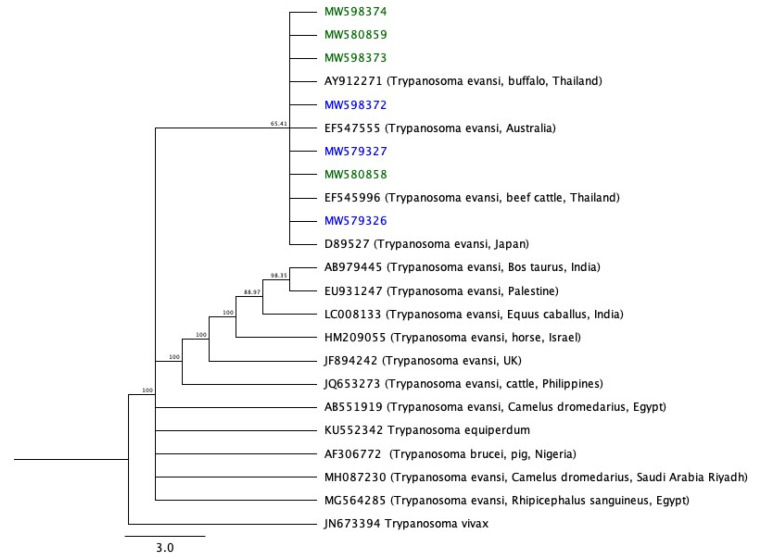
Genetic relationships between samples in the current study and other species. Retrieved from GenBank.

A phylogenetic tree constructed from these sequences is shown in Figure 2. JN673394 (*T. vivax*) was used as the outgroup. The samples from this study are grouped in a clade with *T. evansi* from *C. dromedarius* in Riyadh, Saudi Arabia.

## 4. Discussion

There have been many published studies on the characterization of trypanosomes in general and of *T. evansi* in particular. Among these, the molecular targets that were used are the actin gene [32], the VSG gene [33], and ITS-1 and ITS-2 genes [22,23] of *T. evansi*. The ITS region has been commonly used to identify *T. evansi* [34], and ITS-1 is an appropriate target for the detection of trypanosomes using PCR. Four hundred blood samples were collected from two geographical regions of Saudi Arabia, and 171 samples were successfully amplified. According to this analysis (Table 2), Al-Qassim Province has a higher prevalence of *T. evansi* (46%) than does Riyadh (39.5%). PCR analysis provides higher sensitivity than does microscopic analysis of blood smears and is recommended for the diagnosis of trypanosomiasis [35].

This survey showed a higher prevalence of *T. evansi* in camels in these regions than has been recorded in other surveys from different regions of Saudi Arabia. For instance, 195 camels from Al-Jouf Province (in the northern part of Saudi Arabia) were examined, and 25% of them were infected with *T. evansi* [10]. Other microscopy and serological tests have been used to investigate *T. evansi* infection in camels. Studies that utilized direct thin blood smear testing have produced results showing 2% or 13.2% rates of trypanosomiasis infection in Saudi Arabia as a whole [5,6]. Another study showed a 5.5% rate of infection among camels in the Al-Qassim region [7]. The reported incidence rate of *T. evansi* in camels in different areas of Saudi Arabia varied between 0% and 40% [8]. The prevalence of *T. evansi* infection in Saudi Arabia was 39.4% [9]. In contrast, Alanazi and colleagues [11] reported a higher prevalence: 51% and 49.4% among camels in Riyadh and Al-Qassim, respectively.

Results also varied between neighboring countries. In Iraq, for instance, the prevalence of *Trypanosoma* spp. was confirmed by blood smears to be 31.87% [36]. Studies of *Trypanosoma* spp. in Egypt have shown a prevalence of 4.5%–74.4% [35,37,38,39].

Epidemiological molecular analysis has produced results between 33.9% and 42.1% [18]. These discrepancies may be due to management differences, the climate in which the specimens were collected, or the sample sizes of the tested animals.

The results of this study show no significant difference between the prevalence of *T. evansi* in Riyadh and Al-Qassim. This outcome is contrary to other results from Saudi Arabia [8,10] and from other countries [21,35]. This study’s results also show no significant influence by sex on the prevalence of *T. evansi*, which is consistent with some prior studies [21,35] and inconsistent with others [6,36] that reported male camels had a higher rate of infection than did females in both Saudi Arabia and Iraq. In addition, the present results show no significant effect of age on the rate of *T. evansi* infection. These results disagree with several previous studies [11,21,36]. The high infection rate in adult female camels may be due to several factors, such as travelling through areas where the vector burden is high, low management, stress, drought, insufficient grass, and favoritism by biting flies [21].

Each *Trypanosoma* spp. has unique ITS-1 PCR lengths that were the basis for differentiation between different *Trypanosoma* spp. [11,15,16]. The ITS-1 PCR product size is 250 bp, 400 bp, and 480 bp for the *T. vivax*, *T. simiae*, and *T. brucei* subspecies, respectively, and 700 bp for *T. congolense* savannah.

The phylogenetic analysis of ITS-1 sequences using Geneious Prime Build 2020-04-07 08:42 disclosed a rooted tree with *T. vivax* ITS-1 as an outgroup. The seven Saudi Arabian *T. evansi* isolates have been grouped into the same clade with *T. evansi* isolate from Australia [40], beef cattle (LAM19) identified in Thailand [41], Japanese isolates, and Thailand buffalo (B15). This clade is contained within a larger clade that includes *T. evansi* isolates identified in Egypt from dromedary camels, *C. dromedarius* [23], and from the same in Riyadh, Saudi Arabia [11], *T. brucei* from pigs in Nigeria [42], and *T. evansi* isolates SANTE-FANO-HS from ticks infesting *Canis lupus familiaris* in Egypt. This study indicates that the ancestral root of *T. evansi* is *T. brucei*, a result that has been obtained in similar prior studies [43,44]. Previous research into *T. evansi* has shown that PCR is a more specific, sensitive, and accurate method for the detection of *T. evansi* in camel blood samples than are other approaches that have previously been used in Saudi Arabia. However, in samples from *C. dromedarius* in Riyadh, Saudi Arabia (accession number MH087230) [11], tests were unable to determine the inter- and intraspecific genetic diversity of the parasites. Our samples were grouped into one clade with *T. evansi*.

The limitation of this study lies in using only the ITS1- PCR for testing of the samples, which is general and not specific for *T. evansi* (*T. evansi* type B). Therefore, RoTat 1.2 VSG-PCR is recommended for specific trypanosomiasis detection (*T. evansi* type A). In addition, only seven samples were randomly selected from the positive samples for sequencing and phylogenetic analysis. More samples should be investigated in future studies.

## 5. Conclusions

This study reported that PCR is a more specific, sensitive, and reliable tool for the detection of *T. evansi* in camel blood samples than are the other methods previously used in Saudi Arabia. The results demonstrate the necessity for more detailed phylogenetic analyses and identification of *Trypanosoma evansi* with more taxa and molecular markers. For instance, amplification of the RoTat 1.2 VSG gene should be performed in order to further distinguish among the closely related *Trypanosoma* spp.

## Figures and Tables

**Table 1:** Primers used to amplify the ITS-1 gene using DNA extracted from *T. evansi*.

Gene	Primers	Sequences	References
ITS-1	ITS-1 CF	5′-CCGGAAGTTCACCGATATTG-3′	[16]
	ITS-1 BR	5′-TGCTGCGTTCTTCAACGAA-3′	

**Table 2:** Prevalence of *T. evansi* in camels from Riyadh and Al-Qassim.

Region *	No.	Microscopic Examination	PCR (ITS-1)
Riyadh	200	0:00 (0%)	79 (39.5%)
Al-Qassim	200	0:00 (0%)	92 (46%)

* Collection sites were grouped according to the geographic region of the stocks used in this study. There was no significant difference (*p* ≤ 0.05) between Riyadh and Al-Qassim in the prevalence of *T. evansi.* ITS-1: internal transcribed spacer 1 gene.

**Table 3:** Prevalence of *T. evansi* among different categories of camels.

Categories	Riyadh			Al-Qassim		
	No. of Examined	No. of Infected	% of Infected	No. of Examined	No. of Infected	% of Infected
Young male camels	22	12	54.5%	25	15	60%
Adult male camels	45	17	37.80%	46	17	37%
Young female camels	52	21	40.40%	49	20	40.80%
Adult female camels	81	29	35.80%	80	40	50%
χ2	The chi-square statistic is 2.62. The *p*-value is 0.45. The result is not significant at *p* ≤ 0.05.			The chi-square statistic is 4.53. The *p*-value is 0.21 The result is not significant at *p* ≤ 0.05.		

## Data Availability

All important data are presented in this document.
